# [^18^F]FDG-PET/CT-based risk stratification in women with locally advanced uterine cervical cancer

**DOI:** 10.1186/s12885-024-12232-7

**Published:** 2024-04-23

**Authors:** J.A. Adam, E. Poel, B.L.F. van Eck Smit, C.H. Mom, L.J.A. Stalpers, J.J. Laan, E. Kidd, J. Stoker, S. Bipat

**Affiliations:** 1 Department of Radiology and Nuclear Medicine, Amsterdam UMC, University of Amsterdam, Amsterdam, Netherlands; 2 Department of Experimental Cardiology, Amsterdam UMC, University of Amsterdam, Amsterdam , Netherlands; 3 Department of Gynecologic Oncology, Centre for Gynecologic Oncology Amsterdam Amsterdam UMC, University of Amsterdam, Amsterdam, Netherlands; 4 Department of Radiotherapy, Amsterdam UMC, University of Amsterdam, Amsterdam, Netherlands; 5 Department of Radiation Oncology, Stanford University School of Medicine, Stanford, CA USA

**Keywords:** Prognosis, Locally advanced cervical cancer, Positron emission tomography computed tomography (PET/CT), Metabolic tumor volume (MTV), Maximum standardized uptake value (SUV_max_)

## Abstract

**Background:**

[^18^F]FDG-PET/CT is used for staging and treatment planning in patients with locally advanced cervical cancer (LACC). We studied if a PET-based prediction model could provide additional risk stratification beyond International Federation of Gynaecology and Obstetrics (FIGO) staging in our population with LACC to aid treatment decision making.

**Methods:**

In total, 183 patients with LACC treated with chemoradiation between 2013 and 2018 were included. Patients were treated according to FIGO 2009 and retrospectively reclassified according to FIGO 2018 staging system. After validation of an existing PET-based prediction model, the predicted recurrent free survival (RFS), disease specific survival (DSS) and overall survival (OS) at 1, 3, and 5 years, based on metabolic tumor volume (MTV), maximum standardized uptake value (SUV_max_) and highest level of [^18^F]FDG-positive node was calculated. Then the observed survival was compared to the predicted survival. An area under the curve (AUC) close to or higher than 0.7 was considered adequate for accurate prediction. The Youden (J) index defined survival chance cutoff values for low and high risk groups.

**Results:**

All AUC values for the comparison between predicted and observed outcomes were > 0.7 except for 5-year RFS and for 5-year OS which were close to 0.7 (0.684 and 0.650 respectively). Cutoff values for low and high risk survival chance were 0.44 for the 3-year RFS and 0.47 for the 5-year OS. The FIGO 2009 system could not differentiate between the risk profiles. After reclassification according to FIGO 2018, all patients with stage IIIC2 and IVB fell in the high risk and almost all patients with stages IB2-IIIB and IVA in the low risk group. In patients with stage IIIC1 disease the FIGO stage cannot discriminate between the risk profiles.

**Conclusions:**

Low and high risk patients with LACC can be identified with the PET-based prediction model. In particular patients with stage IIIC1 need additional risk stratification besides the FIGO 2018 staging. The Kidd model could be a useful tool to aid treatment decision making in these patients. Our results also support the choice of [^18^F]FDG-PET/CT imaging in patients with LACC.

**Supplementary Information:**

The online version contains supplementary material available at 10.1186/s12885-024-12232-7.

## Background

Cervical cancer is the third most common cancer in women. In 2021 341,831 women died from the disease worldwide [1, 2]. Despite screening programs and vaccination, a substantial number of patients still present with locally advanced disease (LACC), International Federation of Gynaecology and Obstetrics (FIGO) 2009 stages IB2, IIB–IVA and FIGO 2018 stages IB3– IVA. Patients with LACC are treated with (chemo)radiation. In general, the radiotherapy treatment plan is based on physical examination (under anaesthesia) and imaging.

Staging is done according to the FIGO staging system and this was, until a couple of years ago, mainly based on clinical parameters [3]. Imaging was only added to the latest FIGO staging update in 2018 [4, 5].

2-deoxy-2-[^18^F]fluoro-D-glucose positron emission tomography computed tomography ([^18^F]FDG-PET/CT) is essential in staging LACC, as it has a better performance in showing lymph node and distant metastases compared to magnetic resonance imaging (MRI) or computed tomography (CT) [6]. If [^18^F]FDG-positive lymph nodes are present, patients have a worse prognosis compared to patients with the same clinical FIGO 2009 stage, but [^18^F]FDG-negative nodes [7]. There are, however, only limited data on the change in prognostic ability of FIGO 2018 when additional imaging is taken into account [8].

Considering the role of [^18^F]FDG-PET/CT in LACC, Kidd et al. developed a prediction model based on lymph node status, maximum standardized uptake value (SUV_max_) of the primary tumor, and the metabolic tumor volume (MTV) measured on the PET images [9]. After entering these data into the model, the prediction of recurrence free survival (RFS), disease specific survival (DSS) and overall survival (OS) is expressed as a surviving fraction. Prediction models are often used in the field of oncology. Ideally, a robust prediction model should aid solving a clear clinical problem, be tested on several independent datasets, have mature follow up data and an evaluation of the statistical robustness [10]. However, prediction models are seldom tested using an external patient cohort and rarely specify the choices in treatment regime or cutoff values for a specific treatment choice.

In the treatment of LACC, detection of lymph node metastasis is essential in treatment planning as adjustment of the treatment plan leads to a better survival [11, 12]. Whether to extend the radiotherapy volume to the para-aortic region is a crucial decision, as patients with pelvic node metastasis only have a 5-year survival rate of approximately 60% and it drops to 37% in case of para-aortic metastases [13]. This decision is challenging, as there is chance of (A) false negative nodes (micro-metastases not shown on imaging) [13–16] and (B) false positive, reactive nodes (due to tumor necrosis) [17, 18]. Both overtreatment and under treatment have serious consequences: irradiation of a reactive node means substantial toxicity without survival benefit, while not adequately treating a tumor-positive node could result in impaired survival. Therefore, a risk stratification tool identifying low and high risk patients could aid decision making towards or against a treatment regime with increased toxicity, such as para-aortic radiotherapy.

The purpose of our study to determine risk profiles in locally advanced uterine cervical cancer, based on evaluation of pre-treatment PET-derived characteristics. First, we evaluated an existing PET-based prediction model [9]in our population. After that, in order to study if the prediction model was valid in current practice, patients were retrospectively re-staged according to the FIGO 2018 staging system. Low and high risk groups were defined based on the calculated survival cutoff values.

## Materials and methods

### Patients

Consecutive patients with suspected LACC and intended curative chemoradiation between 1 January 2013 and 31 November 2018 at the Amsterdam University Medical Center (UMC) were included in this retrospective study. All included patients underwent [^18^F]FDG-PET/CT imaging for staging and radiation treatment planning. Exclusion criteria were: unexpected distant metastases or insufficient tumor to background ratio for clear delineation of the tumor volume and no received treatment. Due to the retrospective manner of the study, informed consent was waived by the medical ethical committee of the Amsterdam UMC.

All patients underwent physical examination under anesthesia, including cystoscopy; MRI and [^18^F]FDG-PET/CT imaging before starting (chemo)radiotherapy treatment. Tumor stage was clinically determined according to the FIGO 2009 staging system [3]. During the study all patients were retrospectively restaged according to the FIGO 2018 staging system [4, 5].

### [^18^F]FDG-PET/CT imaging

[^18^F]FDG-PET/CT scans were performed within three weeks after diagnosis, either in regular or in radiation treatment position after fasting for at least six hours. Patients were orally pre-hydrated 24 h prior, and on the day of the investigation (including 0.7 L diluted oral contrast– Joxithalamate 5% [Telebrix Gastro], Guerbet, Villepinte, France). Based on body mass index (BMI), patients were injected with an intravenous bolus of 180–300 MBq [^18^F]FDG. First, an abdominal CT scan was performed with full bladder followed by PET acquisition for radiotherapy planning purposes. After voiding, a second, low dose or diagnostic CT scan was made from the thigh to the skull base, with administration of i.v. contrast agent (Iopromide [Ultravist 300] Bayer Pharma AG, Berlin, Germany). Then, the second PET acquisition followed. This PET/CT scan was used for staging. When low dose CT was performed, PET/CT images were visually compared to the recent diagnostic CT (performed less than three weeks prior to the PET/CT).

The CT and [^18^F]FDG-PET images were fused and viewed (maximum intensity projection, coronal, sagittal and transversal reconstructions) using a Hermes Hybrid viewer (Nuclear Diagnostics AB, Stockholm, Sweden). The CT part of the investigation was additionally viewed on a picture archiving and communication system (PACS, Agfa Enterprise Imaging, Agfa Healthcare System, Mortsel, Belgium).

### Treatment and follow up

All patients were scheduled for curative radiation therapy. External beam radiotherapy (EBRT) was given to the pelvis [total dose 46–50.4 Gy, 1.8–2.0 Gy per fraction] in combination with weekly cisplatin [40mg/m^2^], or hyperthermia as a substitute for concurrent chemotherapy [19, 20]. Radiotherapy was extended to the para-aortic region, if there were suspicious nodes on PET/CT at or above the level of the common iliac vessels. Fletcher brachytherapy to the primary tumor followed during two pulsed dose rate (PRD) applications to a dose of 14 Gy in an hour (total dose 28 Gy). Additional EBRT boost to suspicious parametria and lymph nodes on imaging was given as an integrated boost during pelvic EBRT, or sequentially after brachytherapy up to a biologically equivalent total dose of 60 Gy. All patients had EBRT using an organ sparing technique, (intensity modulated radiotherapy [IMRT] or volumetric modulated arc therapy [VMAT]).

Patients with [^18^F]FDG-positive supraclavicular nodes received platinum based palliative chemotherapy, followed by radiotherapy to the pelvis (typically 30 Gy in 3 Gy fractions). A few patients chose to undergo radiotherapy only. Standard follow up consisted of alternating visits to the gynecologist and radiation oncologist every 3 months for the first two years, every 6 months in the third and fourth years and once in the fifth year. Imaging was only performed during follow up if there was suspicion of recurrent disease, according to local protocols.

### Outcome measures

RFS was defined as time from the day of pathological diagnosis until the date of any recurrent disease, determined by either physical examination, imaging or pathological confirmation. DSS was defined as the time from the date of diagnosis until the date of death caused by cervical cancer. OS was defined as time from the date of diagnosis until the date of death, irrespective of the cause of death. The survival data were collected from the electronic patient records, patients were censored at the date of their last visit without event.

### Data extraction

[^18^F]FDG-PET/CT investigations at diagnosis were analyzed by one of two experienced nuclear medicine physicians (JA 18 years and BE 15 years of experience in PET/CT reading). In case of uncertainties, consensus between the two readers was reached.

### Metabolic tumor volume and SUV_max_

Metabolic tumor volume and SUV_max_ were determined as described earlier [9, 21]. Briefly: first, the bladder activity was masked by creating an automated volume of interest (VOI) by volume rendering and manually adjusted if necessary. Then, an automated VOI was created including the tumor using a fixed 30% threshold at a lowest SUV_max_ limit of 4.0 and adjusted for best fit (mostly 20–40% threshold). The automated region was manually corrected to prevent erroneously inclusion of adjacent tissue– such as ureter, ovary, bowel or non-masked thin border of the bladder. The necrotic part of the tumor was not included in the VOI.

The interobserver variability of the two observers was determined in a subset of thirty scans. The mean difference ± SD of tumor volume and SUV_max_ in this subset was compared. A difference of less than 30% was accepted for measuring metabolic tumor volume, as described before [22]. No difference in the SUV_max_ was accepted.

After analyzing the results of the interobserver variability, the whole cohort (inclusive the subset of thirty scans) was analyzed by either one of the two observers or in case of uncertainty (e.g. low SUV_max_ or necrosis) consensus was reached between the two readers.

The PET-derived tumor volume, the SUV_max_ within the VOI, and the used threshold were recorded on the case report form (CRF).

### Lymph node status

Lymph node status was collected from the [^18^F]FDG-PET/CT report in the electronic patient chart. A lymph node was considered positive if its short axis diameter was more than 1.0 cm, and the [^18^F]FDG-uptake was more than the adjacent vessel or surrounding tissue, or a short axis diameter of less than 1.0 cm in case of very intense uptake (more than twice the adjacent vessel or surrounding tissue) as described before [9]. As patients with positive nodes at the level of the common iliac vessels received para-aortic irradiation, common iliac nodes were considered para-aortic in our cohort.

### Prediction model validation

First, we externally validated an existing [^18^F]FDG-PET/CT-based prediction model to see if it was applicable in our patient cohort treated with contemporary radiotherapy treatment methods.

The validation of the [^18^F]FDG-PET/CT based prediction model by Kidd et al. [9] was performed as follows. The original Cox proportional hazards equations with lymph node status, SUV_max_ and MTV of the primary tumor and of the highest [^18^F]FDG-positive lymph node, and baseline hazards at 12, 36 and 64 months were received after request and were used for the calculation of the estimated survival. The regression parameters, baseline Hazard estimates and prediction models for 1, 3 and 5 years recurrent free survival, disease specific survival and overall survival of the Kidd model are included in the electronic supplementary material.

Then the estimated and observed survival was compared: 1, 3, and 5-year RFS, DSS and OS receiver operating characteristic (ROC) analysis was performed and areas under the curve (AUC) with 95% confidence interval (CI) were calculated. An AUC close to or higher than 0.7 was considered as adequate for a sufficient prediction accuracy as described before [23, 24].

### Risk stratification: low and high risk groups

From the nine studied outcome measures we chose the 3-year RFS for determining “low” and “high” risk groups for deciding on treatment options e.g. para-aortic irradiation, as recurrent disease mostly occurs within 3 years after diagnosis [25]. In addition, we studied the 5-year overall survival as a general accustomed oncological outcome measure.

The optimal cutoff value was calculated using the Youden (J) index of the ROC curve [26]. The J index combines sensitivity and specificity in a single measure, and is used for determining a decision threshold based on the maximized sum of sensitivity and specificity [27], expressed as a chance for an event and translated to survival chance as (1-J). Patients with outcomes above this value were considered as low risk and under as having high risk. Of each model a Kaplan-Meier analysis was performed and differences between low and high risk patients were tested with a log rank test. Statistical analysis was performed with Rstudio (www.r-project.org version 1.2.335).

After the cutoff value was determined we studied the distribution of patients according to FIGO 2009 and 2018 stages in the low and high risk groups.

## Results

### Patients and tumor characteristics

From an initial cohort of 202 patients with LACC who underwent [^18^F]FDG-PET/CT, 19 patients were excluded: 6 had distant metastases, 8 had a different tumor type: vaginal carcinoma, sigmoid adenocarcinoma, melanoma, uterine endometrioid adenocarcinoma, uterine carcinosarcoma, 4 had an insufficient tumor to background ratio for clear delineation of the tumor volume and one patient refused treatment. This resulted in a study cohort of 183 patients.

Patient characteristics and tumor types of our cohort and the cohort analyzed by Kidd et al. and the retrospectively allocated FIGO 2018 stage are summarized in Table 1. Our cohort was comparable to the original cohort, except that we did not include patients with FIGO 2009 stage IB1 as we intended to use the prediction model in LACC. Patients with supraclavicular metastasis only were classified as IVB in our cohort.

**Table 1:** Patient characteristics and tumor pathology (n = 183) [^*^average]

	Studied cohort (n = 183)	Kidd cohort (n = 234)
**Age (years) median [range]**	53.91 [22.09–87.74]	52^*^ [24–94]
**Follow up (months) median [range]**	44.91 [1.87-101.52]	40.7^*^ [5-125]
**FIGO 2009 stage**		
IB2	32 (17.5%)	36 (15%)
IIA	19 (10.3%)	5 (2%)
IIB	69 (37.7%)	102 (43.6%)
IIIA	7 (3.8%)	3 (1.3%)
IIIB	39 (21.3%)	56 (23.9%)
IVA	11 (6.0%)	3 (1.3%)
IVB	6 (3.3%)	0
**FIGO 2018 stage**		
IB2	2 (1.1%)	n.a.
IB3	7 (3.8%)	n.a.
IIA	10 (5.5%)	n.a.
IIB	34 (18.6%)	n.a.
IIIA	4 (2.2%)	n.a.
IIIB	15 (8.2%)	n.a.
IIIC1	68 (37.1%)	n.a.
IIIC2	32 (17.5%)	n.a.
IVA	4 (2.2%)	n.a.
IVB	7 (3.8%)	n.a.
**Histology**		
Squamous cell carcinoma (SCC)	154 (84.2%)	207 (88%)
Adenocarcinoma	17 (9.3%)	16 (7%)
Adenosquamous carcinoma	5 (2.7%)	4 (2%)
Other	7 (3.8%)	7 (3%)

### Interobserver variability

After automated and adjusted delineation, the mean difference of both observers for the MTV was 15.9 ± 14.2%. No difference in SUV_max_ was noted. In 3/30 of the patients the difference between tumor volume was more than 30%. In these cases the tumor was either necrotic or had a low metabolic activity. Therefore it was concluded that in case of a necrotic tumor or a tumor with low metabolic activity, where no automated VOI region with the set threshold could be created, consensus between the two observers should be reached. This was necessary in 19/183 patients (10%).

### Prediction model evaluation

In our cohort, the median MTV was 47.1 cm^3^ [3.0-351.9] and median SUV_max_ was 15.6 [3.7–60.7], in the cohort of Kidd et al. the average MTV of 66.4 cm^3^ [3-535.7] and average SUV_max_ of 12.4 [2.1–50.4]. The level of the highest [^18^F]FDG-positive lymph node was pelvic in 37.2%, para-aortic in 17.5% and supraclavicular in 4.4% of the patients in our cohort. In the cohort of Kidd et al. it was 53%, 18% and 4% respectively. No [^18^F]FDG-positive nodes were seen in 40.9% of the patients compared to 47% in the cohort of Kidd et al. Data are shown in Table 2.

In our cohort, the observed RFS at 1, 3 and 5 years were 0.87, 0.79 and 0.73, the DSS 0.92, 0.85 and 0.76, and OS 0.91, 0.81 and 0.68, respectively.

**Table 2:** PET-derived parameters and treatment (n = 183) [^*^average]

				Studied cohort (n = 183)			Kidd cohort (n = 234)
**Metabolic tumor volume (cm** ^**3**^ **) Median [range]**				47.04 [3.01-351.88]			47.07^*^ [3.01-351.88]
**Tumor SUV**_**max**_ **Median [range]**				15.57 [3.7-60.69]			12.4^*^ [2.1–50.4]
**Highest [** ^**18**^ **F]FDG-positive lymph node**							
No suspicious nodes				75 (40.9%)			109 (46.6%)
Pelvic				68 (37.2%)			84 (35.9%)
Para-aortic				32 (17.5%)			31 (13.2%)
Supraclaviculair				8 (4.4%)			10 (4.3%)
**Treatment**							
Chemoradiation				137 (74.9%)			90%
Radiation with hyperthermia				33 (18.0%)			none
Radiation therapy only				9 (4.9%)			10%
Palliatieve chemotherapy and radiotherapy				4 (2.2%)			none

*Abbreviations* SUV_max_– maximum standardized uptake value, [^18^F]FDG– 2-deoxy-2-[^18^F]fluoro-D-glucose

Comparison of the observed survival with the model-based survival estimates yielded AUCs between 0.7 and 0.807 for all outcomes except for 5-year RFS (0.684) and 5-year OS (0.650), the detailed data including the confidence intervals are shown in Table 3.

Considering that the majority of the patients had squamous cell carcinoma (84.1%), we also calculated the AUC for this group only. The AUC was somewhat higher for patients with squamous cell carcinoma compared to the whole cohort for all studied survival categories, see Table 3.

**Table 3:** Area under the curve and 95% confidence intervals for the estimated versus observed survival in the whole patient group (n = 183) and in patients with squamous cell carcinoma (n = 154)

Whole cohort (n = 183)	RFS [95% CI]	DSS [95% CI]	OS [95% CI]
1-year	0.744 [0.633–0.855]	0.806 [0.669–0.943]	0.807 [0.690–0.925]
3-year	0.710 [0.617–0.802]	0.779 [0.678–0.881]	0.762 [0.670–0.854]
5-year	0.684 [0.595–0.774]	0.702 [0.606–0.798]	0.650 [0.562–0.737]
**Squamous cell carcinoma (SCC)** (*n* = 154)			
1-year	0.762 [0.636–0.887]	0.840 [0.722–0.958]	0.836 [0.744–0.928]
3-year	0.736 [0.635–0.836]	0.793 [0.679–0.906]	0.773 [0.677–0.868]
5-year	0.734 [0.643–0.825]	0.759 [0.658–0.859]	0.680 [0.586–0.774]

*Abbreviations* RFS– recurrence free survival, DSS– disease specific survival, OS– overall survival

#### Risk stratification

**Fig. 1:**
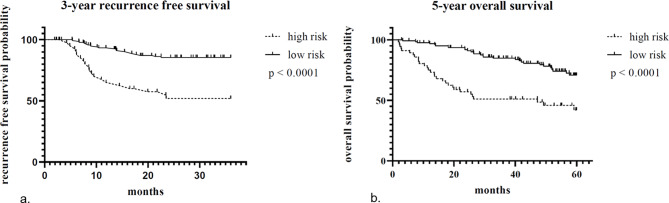
(**a**) Kaplan-Meyer curves of patients with low and high risk for 3-year RFS. (**b**) Kaplan-Meyer curves of patients with low and high risk for 5-year OS

When determining the cutoff value for low and high risk patients, the best fit for the 3-year RFS appeared at a cutoff value of survival chance of 0.43 (Fig. 1a) and for the 5-year OS of 0.47 (Fig. 1b).

**Fig. 2:**
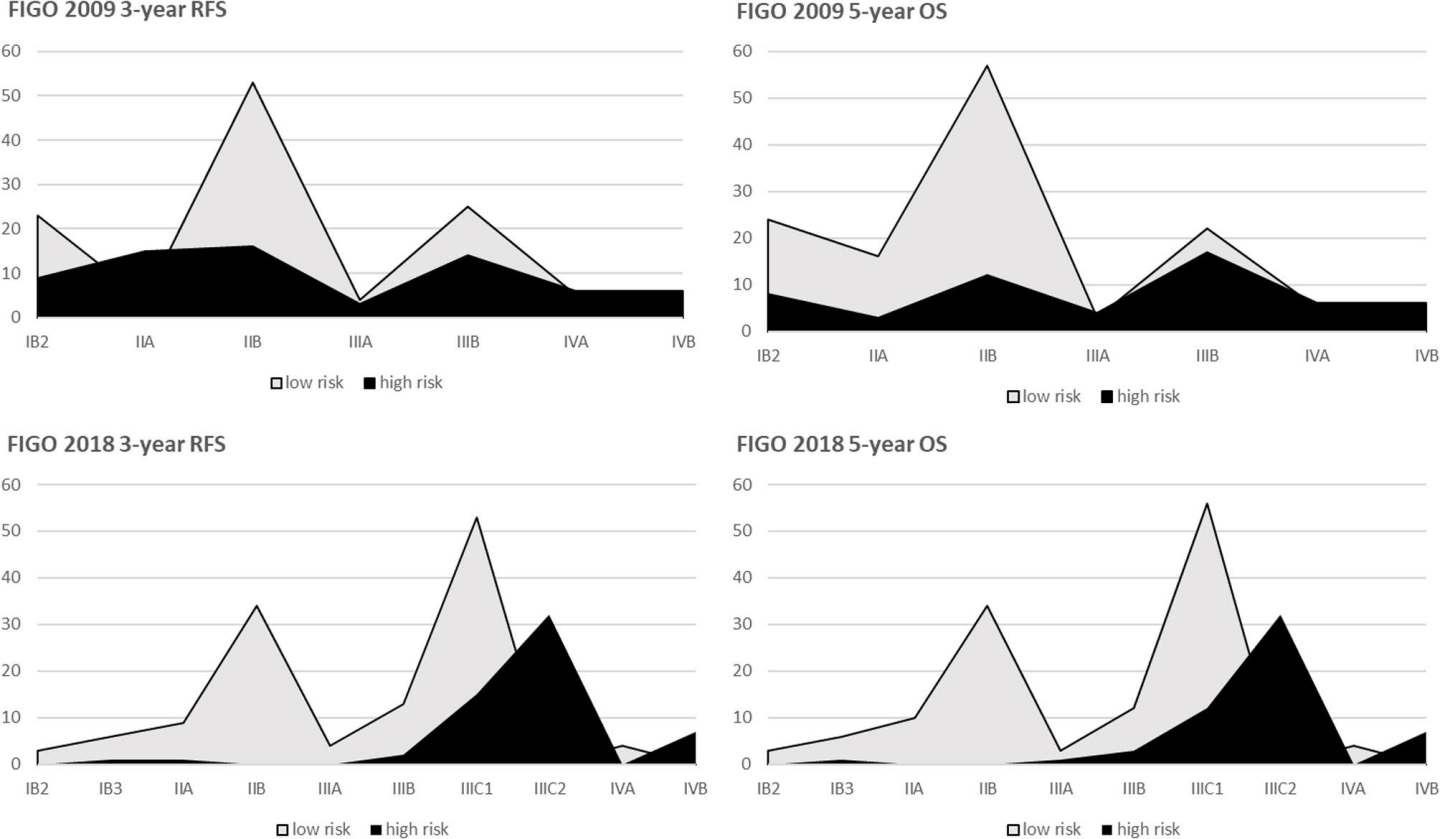
Distribution of patients with stages FIGO 2009 and 2018 LACC in the low and high risk group determined by the Kidd prediction model for 3-year RFS and 5-year OS. *Abbreviations*: RFS– recurrent free survival, OS– overall survival, LACC– locally advanced cervical cancer

When dividing patients into low or high-risk groups, all the patients in the high risk group had [^18^F]FDG-positive para-aortic or supraclavicular nodes. None of the patients in the low risk group showed [^18^F]FDG-positive nodes outside the pelvis. The distribution of the FIGO 2009 stage was similar with representation of all FIGO stages in both risk groups (Table 4). After patients were restaged according to the FIGO 2018 stage, the distribution had changed. As expected, all patients with [^18^F]FDG-positive para-aortic nodes (stage IIIC2) fell into the high risk group. In stages IB2–IIIA and IIIB the majority of the patients fell in the low risk group for both outcomes. In case of patients with stage IIIC1 disease, however, the FIGO 2018 stage cannot properly differentiate between low and high risk patients (Table 4; Fig. 2). These observations were valid for all studied outcome measures (data not shown).

**Table 4:** Characteristics of high and low risk patients for 3-year recurrence free survival and 5-year overall survival

	3-year RFS		5-year OS	
	High risk (n = 58)	Low risk (n = 125)	High risk (n = 56)	Low risk (n = 127)
**Age [years]**	49.3 [22.1–82.9]	55.71 [26.9–87.7]	50.2 [22.1–82.9]	55.5 [26.9–87.7]
**MTV [cm** ^**3**^ **]**	71.0 [12.6-351.9]	36.1 [3.0-214.7]	83.8 [12.6-351.9]	36.1 [3.0-144.4]
**Tumor SUV**_**max**_	19.61 [9.3–60.7]	14.3 [3.67–29.4]	18.0 [9.3–60.7]	14.4 [3.7–51.1]
**[** ^**18**^ **F]FDG-positive nodes**				
No suspicious nodes	3 (5.2%)	72 (57.6%)	4 (7.1%)	71 (55.9%)
Pelvic	15 (25.9%)	53 (42.4%)	12 (21.4%)	56 (44.1%)
Para-aortic	32 (55.2%)	0	32 (57.1%)	0
Supraclaviculair	8 (13.8%)	0	8 (14.3%)	0
**FIGO 2009 stage**				
IB2	9 (15.5%)	23 (18.4%)	8 (14.3%)	24 (18.9%)
IIA	4 (6.9%)	15 (12.0%)	3 (5.3%)	16 (12.6%)
IIB	16 (27.6%)	53 (42.4%)	12 (21.4%)	57 (44.9%)
IIIA	3 (5.2%)	4 (3.2%)	4 (7.1%)	3 (2.4%)
IIIB	14 (24.1%)	25 (20%)	17 (30.3%)	22 (17.3%)
IVA	6 (10.3%)	5 (4.0%)	6 (10.7%)	5 (3.9%)
IVB	6 (10.3%)	0	6 (10.7%)	0
**FIGO 2018 stage**				
IB2	0	2 (1.6%)	0	2 (1.6%)
IB3	1 (1.7%)	6 (4.8%)	1 (1.8%)	6 (4.7%)
IIA	1 (1.7%)	9 (7.2%)	0	10 (7.9%)
IIB	0	34 (27.2%)	0	34 (26.8%)
IIIA	0	4 (3.2%)	1 (1.8%)	3 (2.4%)
IIIB	2 (3.4%)	13 (10.4%)	3 (5.3%)	12 (9.4%)
IIIC1	15 (25.9%)	53 (42.4%)	12 (21.4%)	56 (44.1%)
IIIC2	32 (55.2%)	0	32 (57.1%)	0
IVA	0	4 (3.2%)	0	4 (3.1%)
IVB	7 (12.1%)	0	7 (12.5%)	0

*Abbreviations* RFS– recurrent free survival, OS– overall survival, MTV– metabolic tumor volume, SUV_max_– maximum standardized uptake value, [^18^F]FDG– 2-deoxy-2-[^18^F]fluoro-D-glucose

## Discussion

To be able to define a low risk and high risk group for recurrence in LACC, we have tested the accuracy of an earlier described PET-based prediction model in our retrospective cohort of 183 consecutive patients with LACC. The observed survival was in line with the earlier reported survival in LACC [28]. When comparing the estimated survival with the observed survival, the predictive accuracy was sufficient to predict 1-, 3- and 5-year RSF, DSS and OS with AUC values between 0.650 and 0.807. When looking at patients with squamous cell carcinoma only, the AUC values were slightly higher and had a range of 0.680–0.840. These results indicate that the Kidd prediction model is applicable to our patient population with LACC.

Lora et al. previously externally validated the Kidd prediction model [29] and concluded that the 3-year OS and the 1-year DSS are statistically valid with an AUC of 0.69 and 0.64 respectively. Although the observed survival of the patient cohort of Lora et al. was similar to ours, the prediction model performed better in our cohort. This could be explained by the fact that the validation of Lora et al. is based on the visual nomogram and not on a Cox-analysis of the prediction model. Second, in the patient cohort of Lora et al. all patients with bulky disease or FIGO 2009 stage ≥ IIB received para-aortic irradiation irrespective of the imaging results. Similar to Kidd et al., in our cohort, para-aortic radiotherapy was only applied in case of suspicious (e.g. [^18^F]FDG-positive) para-aortic nodes.

One of the challenging situations in LACC is the decision to extend the radiation volume to the para-aortic region. Considering that most recurrences occur within three years, we used the 3-year RFS to evaluate whether the Kidd prediction model could aid the decision for extending the radiotherapy field. Our results suggest that the 3-year RFS at a predicted survival chance cutoff value of 0.43 could differentiate between high and low risk patients. When looking at the risk profile of the low and high risk patients, all patients with para-aortic nodes (FIGO 2018 stage IIIC2) fall into the high risk category. This prognostic high risk suggests that in these patients the chance that the nodes are false positive on imaging is small and decision should be towards irradiation while accepting toxicity. This is in line with our earlier data that the certainty of an [^18^F]FDG-positive node improves with prevalence [30] and the earlier reported finding that patients with [^18^F]FDG-positive nodes had worse prognosis than patients with negative nodes in the same FIGO 2009 stage [7]. Another application of the prediction model could be to select high risk patients for additional treatment strategies. In this case, patients with the calculated 5-year OS chance under the cutoff of 0.47 should be considered high risk and potentially benefit from for example imaging during follow up, experimental immune- or targeted therapy.

When looking at the FIGO 2009 stages, we would expect that the high risk group contains more patients with higher FIGO stages. However, all FIGO 2009 stages were represented in both risk categories. This means that the FIGO 2009 stage alone is not sufficient to stratify the risk of patients with LACC, which has already been acknowledged by adding imaging to the FIGO 2018 staging. After patients were retrospectively restaged according to the FIGO 2018 stage, the distribution changed to a more skewed pattern where the risk profile was more in line with the FIGO stage. In case of patients with stage IIIC1 disease however, the FIGO 2018 stage cannot properly differentiate between low and high risk. Especially in these patients treatment choice could be optimized with the use of the Kidd prediction model. Considering that the choice of imaging has been left open in FIGO 2018, our results suggest that [^18^F]FDG-PET/CT should be performed in LACC, as PET-based parameters are a useful addition in risk stratification.

Our data provide additional value as prediction models should be tested on several independent datasets [10]. In addition, the study of Lora et al. [29] included patients treated 1999–2014, the study of Kidd et al. [9] patients treated in the period 1998–2008 and the patients in our cohort between 2013 and 2018. The patients in our cohort received treatment with more contemporary radiotherapy techniques (e.g. IMRT and VMAT), allowing smaller planning target volume margins and simultaneous integrated protection. Furthermore we showed that the model is applicable in FIGO 2018 staging system.

There are limitations to our study. First, the retrospective nature. Second, our cohort is relatively small, with less than the recommended 100 number of events [31], and a small group per stage. However, the sample size is comparable to the earlier reported external prediction model validations in LACC. We included all tumor types, while other tumor types, e.g. adenocarcinoma could have different outcomes than squamous cell carcinoma in LACC [32]. Indeed, our study show that the prediction model performs slightly better in case of SCC than in the whole group, nevertheless the model is robust enough in all tumor types of LACC. We included patients with supraclavicular nodes (stage IVB). This is arguable, as these patients have the worst prognosis in LACC, and inclusion might worsen the robustness of the prediction model. However, the original model is based on the presence of metastases in supraclavicular nodes as well.

## Conclusion

In this study we show that an existing prediction model is applicable in our patient population with LACC and that it can identify low and high risk patients. Our data suggest that patients with an [^18^F]FDG-positive para-aortic or supraclavicular nodes (FIGO 2018 stage IIIC2 and IVB) belong to the high risk group. This means that when these nodes are present, treatment choice should be towards irradiation and biopsies for pathological confirmation may be considered as superfluous in high risk patients. In our population, particularly in stage IIIC1 the FIGO 2018 staging is not sufficient for risk stratification. The Kidd prediction model could be a useful addition for clinical decision making in these patients. Combining PET-prediction models with clinical parameters or other imaging data could further aid decision making in the future.

### Electronic supplementary material

Below is the link to the electronic supplementary material.

Supplementary Material 1

## Data Availability

The datasets supporting the conclusion of this article are to the utmost extent included within the article and its additional files. The remaining data are available from the corresponding author on reasonable request.

## Abbreviations

AUC: area under the curve
BMI: body mass index
CI: confidence interval
CRF: case report form
CT: computed tomography
DSS: disease specific survival
EBRT: external beam radiotherapy
[^18^F]FDG: 2-deoxy-2-[^18^F]fluoro-D-glucose
FIGO: International Federation of Gynaecology and Obstetrics
IMRT: intensity modulated radiation therapy
J-index: Youden index
LACC: locally advanced cervical cancer
MRI: magnetic resonance imaging
MTV: metabolic tumor volume
OS: overall survival
PACS: picture archiving and communication system
PET: positron emission tomography
PET/CT: positron emission tomography computed tomography
PRD: pulsed dose rate
RFS: recurrence free survival
ROC: receiver operating characteristic
SCC: squamous cell carcinoma
SD: standard deviation
SUV_max_: maximum standardized uptake value
VMAT: volumetric modulated arc therapy
VOI: volume of interest
