# Supplementary material for: [18F]FDG-PET/CT-based risk stratification in women with locally advanced uterine cervical cancer
Source: BMC Cancer. 2024 Apr 23;24:513. doi: 10.1186/s12885-024-12232-7 (PMC11040958; doi:10.1186/s12885-024-12232-7)
Supplement: Supplementary file 1 — Supplementary Material 1 [file 12885_2024_12232_MOESM1_ESM.docx]

**Regression parameters for the studied endpoints**

| Endpoint | Regression parameters | | | | |
| --- | --- | --- | --- | --- | --- |
|  | LN =None | LN=Pelvic | LN=SC | MTV | SUV_max_ |
| RFS | -1.4087 | -1.1534 | 1.8049 | 0.0018 | 0.0326 |
| DSS | -1.3083 | -0.9855 | 1.0649 | 0.0026 | 0.0113 |
| OS | -0.9077 | -0.8335 | 0.8961 | 0.0023 | 0.0089 |

Abbreviations: LN – lymph node, SC – supraclavicular, MTV – metabolic tumor volume, SUV_max_ – maximum standardized uptake value, RFS – recurrence free survival, DSS – disease specific survival, OS – overall survival

**Baseline hazard estimates**

| **Time (RFS)** | **Hazard (RFS)** | **Time (DSS)** | **Hazard (DSS)** | **Time (OS)** | **Hazard (OS)** |
| --- | --- | --- | --- | --- | --- |
| 0  1  2  3  4  5  6  7  8  9  10  12  13  14  15  17  18  19  23  25  26  27  28  31  32  35  36  37  38  62  64  80 | 0.02657782  0.02969006  0.03310288  0.04004836  0.06303028  0.13391539  0.15945379  0.17011613  0.19749887  0.20883111  0.23262057  0.24642403  0.25354589  0.26088389  0.26840542  0.29845122  0.30902364  0.33078269  0.36604896  0.40383011  0.41667771  0.42982501  0.44379043  0.45964791  0.47574499  0.49453461  0.51402422  0.53428981  0.55616093  0.59216036  0.66617052  0.71162617 | 2  3  4  5  7  8  9  10  11  12  13  14  15  17  18  19  20  21  22  23  24  25  26  28  30  31  34  36  38  48  54  64  67  70 | 0.003067972  0.006158935  0.009381388  0.025988596  0.03672369  0.052258789  0.064527727  0.073081358  0.101604625  0.106534857  0.12215775  0.12784451  0.151958911  0.159048329  0.166283063  0.173702148  0.181200173  0.188806838  0.196614746  0.212645033  0.22956244  0.238092329  0.255321608  0.264755152  0.285898119  0.296946748  0.309108801  0.322676201  0.336698719  0.361293404  0.386376641  0.416458741  0.448638475  0.482036615 | 2  3  4  5  7  8  9  10  11  12  13  14  15  17  18  19  20  21  22  23  24  25  26  28  30  31  34  36  38  48  54  64  67  70  84 | 0.003531621  0.010701006  0.018069865  0.048483781  0.056523893  0.069369823  0.087503284  0.096876237  0.128129396  0.13896423  0.15600891  0.162094195  0.19429769  0.201648356  0.216716107  0.224444273  0.240128864  0.248087225  0.256228347  0.281463789  0.299167672  0.308111635  0.326197134  0.346096523  0.368372292  0.380022408  0.392815859  0.406985318  0.42164268  0.445565392  0.470031784  0.499356235  0.530810822  0.56330322  0.610035383 |

**All regression parameters and baseline hazard (per time points) were provided by Kidd et al.**

For the 5 years regression models we used the baseline hazard at time point 64 months.

Abbreviations: RFS – recurrent free survival, DSS – disease specific survival, OS – overall survival

**Prediction models**

RECURRENCE FREE SURVIVAL (RFS)

1Y RFS🡪 Pred= 0.24642403 -1.4087 * no_ln -1.1534 * pelvic_ln + 1.8049 * sc_ln+ 0.0018 * MTV + 0.0326 * SUV_max_

3Y RFS🡪Pred= 0.51402422 -1.4087 * no_ln -1.1534 * pelvic_ln + 1.8049 * sc_ln+ 0.0018 * MTV + 0.0326 * SUV_max_

5Y RFS🡪 Pred= 0.66617052 -1.4087 *no_ln -1.1534 * pelvic_ln + 1.8049 * sc_ln+ 0.0018 * MTV + 0.0326 *SUV_max_

DISEASE SPECIFIC SURVIVAL (DSS)

1Y DSS🡪Pred= 0.106534857 -1.3083 *no_ln -0.9855 *pelvic_ln + 1.0649 * sc_ln+ 0.0026 * MTV + 0.0113 * SUV_max_

3Y DSS🡪 Pred= 0.322676201 -1.3083 *no_ln -0.9855 *pelvic_ln + 1.0649 * sc_ln+ 0.0026 * MTV + 0.0113 *SUV_max_

5Y DSS🡪 Pred= 0.416458741-1.3083 *no_ln -0.9855 *pelvic_ln + 1.0649 * sc_ln+ 0.0026 * MTV + 0.0113 *SUV_max_

OVERALL SURVIVAL (OS)

1 Y OS🡪 Pred= 0.13896423 -0.9077 *no_ln -0.8335 * pelvic_ln + 0.8961 * sc_ln+ 0.0023 *MTV + 0.0089 *SUV_max_

3Y OS🡪 Pred= 0.406985318 -0.9077 *no_ln -0.8335 *pelvic_ln + 0.8961 *sc_ln+ 0.0023 * MTV + 0.0089 *SUV_max_

5Y OS-🡪 Pred= 0.499356235 -0.9077 *no_ln -0.8335 * pelvic_ln + 0.8961 * sc_ln+ 0.0023 * MTV + 0.0089 * SUV_max_

Event was calculated as follows: $\frac{exp(pred)}{1+\exp\left( pred \right)}$

Abbreviations: Y – year, Pred – predicted, ln – lymph node, sc – supraclaviculair, SUVmax – maximum standardized uptake value
